# DEEP MOVEMENT: Deep learning of movie files for management of endovascular thrombectomy

**DOI:** 10.1007/s00330-023-09478-3

**Published:** 2023-02-27

**Authors:** Brendan Kelly, Mesha Martinez, Huy Do, Joel Hayden, Yuhao Huang, Vivek Yedavalli, Chang Ho, Pearse A. Keane, Ronan Killeen, Aonghus Lawlor, Michael E. Moseley, Kristen W. Yeom, Edward H. Lee

**Affiliations:** 1 Department of Radiology, Stanford University School of Medicine, Stanford, CA USA; 2 Department of Radiology, St Vincent’s University Hospital, Elm Park, Dublin 4 Ireland; 3 Insight Centre for Data Analytics, University College Dublin, Belfield, Dublin 4 Ireland; 4 Department of Clinical Radiology and Imaging Sciences, Indiana University School of Medicine, Indianapolis, IN USA; 5 St. Joseph Mercy, Phoenix, USA; 6 Moorfields Eye Hospital, London, UK; 7 Institute of Ophthalmology, University College London, London, UK

**Keywords:** Radiology, Deep learning, Stroke, Angiography, Thrombectomy

## Abstract

**Objectives:**

Treatment and outcomes of acute stroke have been revolutionised by mechanical thrombectomy. Deep learning has shown great promise in diagnostics but applications in video and interventional radiology lag behind. We aimed to develop a model that takes as input digital subtraction angiography (DSA) videos and classifies the video according to (1) the presence of large vessel occlusion (LVO), (2) the location of the occlusion, and (3) the efficacy of reperfusion.

**Methods:**

All patients who underwent DSA for anterior circulation acute ischaemic stroke between 2012 and 2019 were included. Consecutive normal studies were included to balance classes. An external validation (EV) dataset was collected from another institution. The trained model was also used on DSA videos post mechanical thrombectomy to assess thrombectomy efficacy.

**Results:**

In total, 1024 videos comprising 287 patients were included (44 for EV). Occlusion identification was achieved with 100% sensitivity and 91.67% specificity (EV 91.30% and 81.82%). Accuracy of location classification was 71% for ICA, 84% for M1, and 78% for M2 occlusions (EV 73, 25, and 50%). For post-thrombectomy DSA (*n* = 194), the model identified successful reperfusion with 100%, 88%, and 35% for ICA, M1, and M2 occlusion (EV 89, 88, and 60%). The model could also perform classification of post-intervention videos as mTICI < 3 with an AUC of 0.71.

**Conclusions:**

Our model can successfully identify normal DSA studies from those with LVO and classify thrombectomy outcome and solve a clinical radiology problem with two temporal elements (dynamic video and pre and post intervention).

**Key Points:**

*• DEEP MOVEMENT represents a novel application of a model applied to acute stroke imaging to handle two types of temporal complexity, dynamic video and pre and post intervention.*

*• The model takes as an input digital subtraction angiograms of the anterior cerebral circulation and classifies according to (1) the presence or absence of large vessel occlusion, (2) the location of the occlusion, and (3) the efficacy of thrombectomy.*

*• Potential clinical utility lies in providing decision support via rapid interpretation (pre thrombectomy) and automated objective gradation of thrombectomy outcomes (post thrombectomy).*

**Supplementary Information:**

The online version contains supplementary material available at 10.1007/s00330-023-09478-3.

## Introduction

Ischaemic stroke is a time-dependent disease that remains a significant cause of morbidity and mortality [1]. Stroke is a compelling use case for radiology artificial intelligence (AI) due to its time-sensitive nature. Mechanical thrombectomy to extract the causal intra-arterial thrombus has revolutionised treatment and improved outcomes [2]. Indeed, approximately 1.9 million neurons are lost for every minute a patient is left untreated [3]. Therefore, measures that can increase the speed and efficacy of the diagnostic and treatment process have the potential to improve patient outcomes.

The advent of increased computer processing power has facilitated the use of deep learning computer vision tasks [4]. These methods have reached expert-level performance on several medical imaging tasks [5, 6]. However, while many papers report expert-level results by using deep learning in radiology, most apply only a narrow range of techniques to a narrow selection of use cases [7]. This has led to calls for models that can incorporate prior images [8]. Indeed the development of such models has been called “essential to provide meaningful improvements” in the field [8]. Recently, computer vision for video analysis has attracted research interest [9, 10]. Techniques for object tracking, for example, have useful applications in driverless cars and other growth industries. Medical application of video analysis has been slower to develop, however, although promise has been shown in echocardiography [11, 12] with emerging potential applications in diagnostic radiology [13].

Interventional radiologists interpret medical imaging in real time in a video format in clinical practise, for example in the form of a “run” (time ordered series of images) from digital subtraction angiography (DSA). In the interventional suite, rapid assisted interpretation of these “runs” could help to reduce intervention times by aiding detection and determining the need for re-intervention particularly in challenging or borderline cases.

Our aim was to develop a model that takes as input a video of a cerebral DSA and identify the presence or absence of large vessel occlusion (LVO), locate the level of occlusion, and assess the success of thrombectomy. The proposed task contains two elements which add temporal complexity: there is the dynamic nature of the DSA run that changes frame by frame, and also the change detection element, comparing pre and post-intervention runs.

Our primary hypothesis is that our model could identify occlusions in real time, which would have significant potential clinical utility by providing decision support for rapid interpretation (pre thrombectomy). Our secondary hypothesis is that our model could be applied post thrombectomy as automated objective gradation of outcomes.

## Methods

This manuscript was prepared according to the CLAIM checklist [14]. Our code is available on GitHub (https://github.com/edhlee/DeepMovement).

### Patient selection

This retrospective study was HIPAA compliant, approved by the Stanford School of Medicine IRB and University of Indiana IRB with an approved data sharing agreement between these two institutions. We included all patients who underwent DSA for acute ischaemic stroke in a single tertiary university-affiliated centre from 2012 to 2019. These patients had an abnormal CT angiogram and were referred to neurointerventional radiology. Consecutive patients who underwent DSA for other indications and had a normal study (from 2019) were used as controls. An independent validation cohort from a different university-affiliated tertiary referral centre was also retrospectively collected. Exclusion criteria were those patients without the standard posteroanterior (PA) and lateral projections of the circle of Willis.

### Truth determination

All cerebral angiograms were assessed by board-certified neurointerventional radiologists at the respective centres, all of whom are board-certified and subspecialty trained. The location of occlusion was specified and the post-thrombectomy result was assessed using a structured report. LVO was defined as the blockage of the terminal internal carotid artery (ICA) and M1 (horizontal) or M2 (vertical) segment of the middle cerebral artery (MCA). Modified Treatment in Cerebral Ischaemia (mTICI) scores were recorded. Scores were verified by a separate radiologist for this study. When no score was available, the text of the structured report and the images were reviewed to assign a mTICI score retrospectively. Disagreements were decided by a third certified radiologist.

### Video preparation

DICOM files of DSAs were collated in OsirixMD (version 11) and converted to mp4 format. For the cases where thrombectomy was performed, four videos were created per patient (PA and lateral views, pre and post thrombectomy) while two videos of the normal controls (PA and lateral) were recorded. DICOM files received from the outside institution were loaded onto OsirixMD and processed in the same way.

### Design

The models were trained to classify whether an LVO was present. Each case was classified as normal, ICA, M1, or M2. Next, the post-thrombectomy videos were given as input and were reclassified. Movement between classes (either from proximal to distal occlusion or to normal) was deemed as identification of a successful thrombectomy. Finally, the model was used to classify the post-thrombectomy videos as having an mTICI of 3 (complete antegrade reperfusion) or < 3 (incomplete antegrade reperfusion).

### Deep learning models

We investigated the performance of several deep learning models using different architectures. First, we employed a 2D convolutional neural network (CNN) model (Xception) which uses only single frames, stacked 2D CNN (stacked-Xception) using multiple frames (2.5D), a 2D vision transformer (ViT), and a 3D CNN (Inception 3D) capturing full special and temporal resolution.

**Fig. 1:**
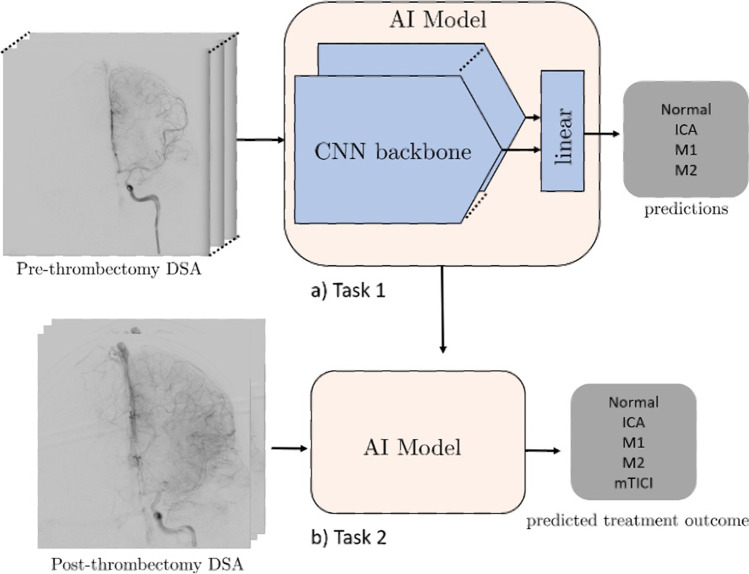
Summary of DEEP MOVEMENT. Our models are trained and evaluated on DSAs of patients before thrombectomy in task 1 (**a**). The stacked-Xception model is shown as an example. The pre-thrombectomy model is next evaluated to predict treatment outcome of post thrombectomy to assess the quality of reperfusion in task 2 (**b**)

On all deep learning approaches except the stacked-Xception + ViT ensemble, we train for 20 epochs with Adam optimizer [15], a learning rate decay of 0.9 every epoch. The ViT component requires considerably longer training times to achieve convergence. For example, training with 20 epochs and 0.9 learning rate decay every epoch results in a training-set F1 score of approximately 0.6 compared to > 0.75 of other models. Data augmentations included horizontal flipping of the frames (for the 2D approaches), cropping, and random brightness distortions. The Xception backbone was pretrained on ImageNet, the inception-3D on Kinetics, and ViT (2D) backbone on Cifar-100. A summary is seen in Fig. 1. All model architectures are detailed in the supplemental GitHub link (https://github.com/edhlee/DeepMovement).

Data augmentations included horizontal flipping of the frames (for the 2D approaches), cropping, and random brightness distortions. Grad-cams (dynamic saliency maps) were produced to aid interpretability.

### Analysis

Our model was trained and evaluated on both an internal independent test set and an external validation set. Identification of LVO was measured using the F1 score, sensitivity, and specificity. Location classification was evaluated using accuracy. The grading of post-thrombectomy results was evaluated using the area under the ROC curve. The same stacked-Xception model was then employed without fine-tuning or additional transfer learning on the external validation data.

## Results

### Sample size

In total, 1024 videos comprising 287 patients were included for analysis, 225 with occlusion and 62 normal, 237 for training, and 50 for testing. Of the 50 for testing, there were 7, 11, 21, and 11 normal, ICA, M1, and M2 occlusions, respectively. Five cases were excluded as the requisite views were not obtained. Three cases were excluded due to file corruption. The median age was 76 and 47% of the included patients were female. Forty-four patients were included for external validation with no cases excluded. Patient demographics are summarised in Table 1.

**Table 1:** Patient demographic information

Local data		
Total number		287
Sex	Male	152
	Female	135
Mean age (range)		76 (18–98)
Side	Left	117
	Right	108
Site	Normal	62
	ICA	60
	M1	116
	M2	49
External data		
Total number		44
Sex	Male	21
	Female	23
Mean age (range)		61 (18–94)
Side	Left	23
	Right	21
Site	Normal	11
	ICA	11
	M1	12
	M2	10

### Results on pre-thrombectomy videos with stacked-Xception

**Fig. 2:**
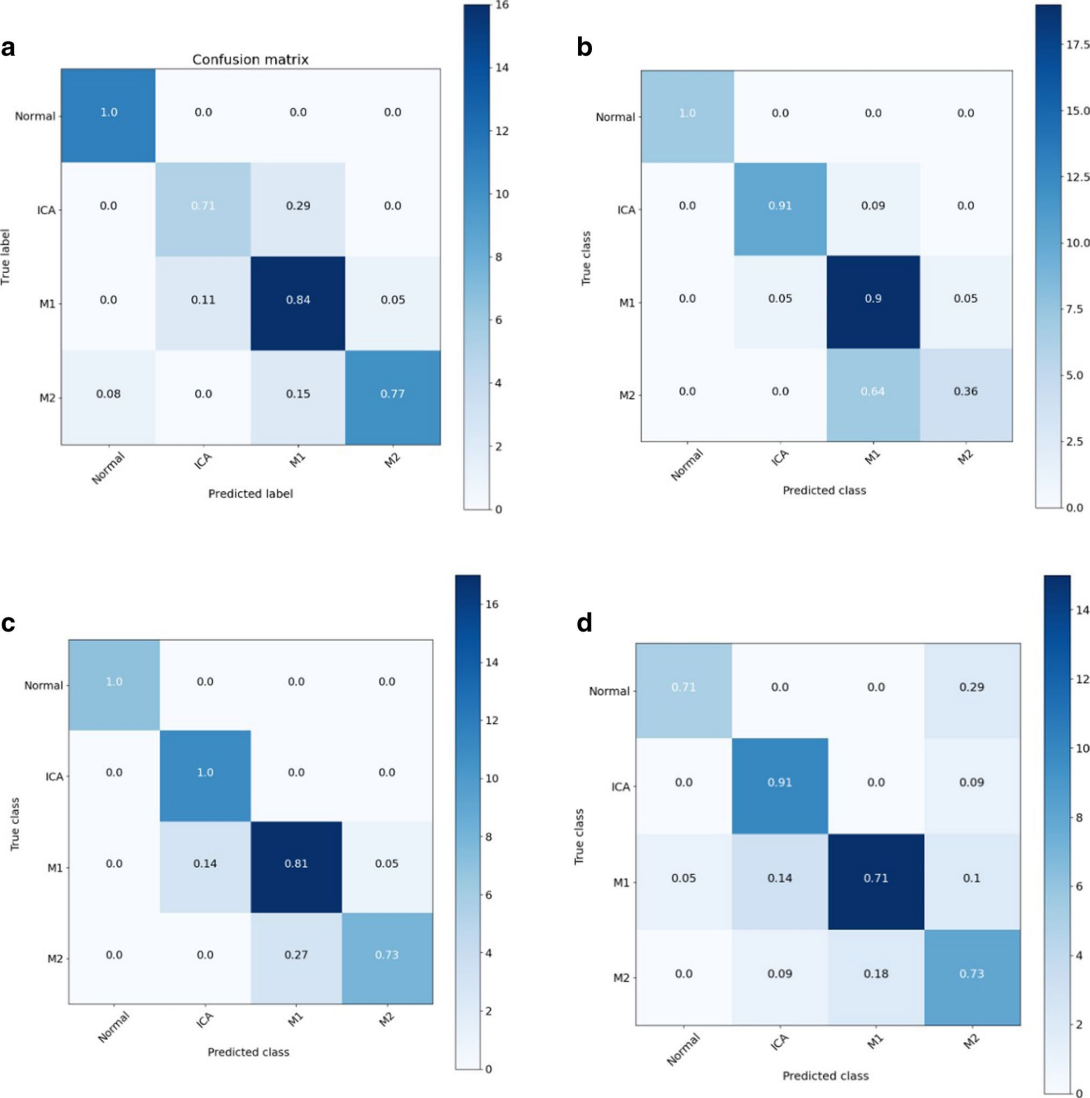
(**a**–**d** Clockwise from top left) Confusion matrix showing results of various models on the Stanford test set: (**a**) stacked-Xception, (**b**) 3D model (inception-3D), (**c**) stacked-Xception + 3D, (**d**) stacked-Xception + ViT

With stacked-Xception, occlusion identification was achieved with 100% sensitivity (CI 90.75 to 100.00%) and 91.67% specificity (CI 61.52 to 99.79%) (Table 2 and Fig. 2a). Accuracy of location classification was 71% for ICA, 84% for M1, and 78% for M2 occlusions.

**Table 2:** Summary of results for stacked-Xception

Location	Task	Site	Sensitivity	Specificity	Accuracy
Internal	Identification of occlusion		100% (CI 90.75 to 100.00%)	91.67% (CI 61.52 to 99.79%)	0.98
	Classification location	ICA			0.71
		M1			0.84
		M2			0.78
	Successful reperfusion	ICA			1.0
		M1			0.88
		M2			0.35
External	Identification of occlusion		91.30% (71.96 to 98.93%)	81.82% (48.22 to 97.72%)	
	Classification location	ICA			0.73
		M1			0.35
		M2			0.50
	Successful reperfusion	ICA			0.89
		M1			0.88
		M2			0.60

### Results on pre-thrombectomy videos with alternative models

**Fig. 3:**
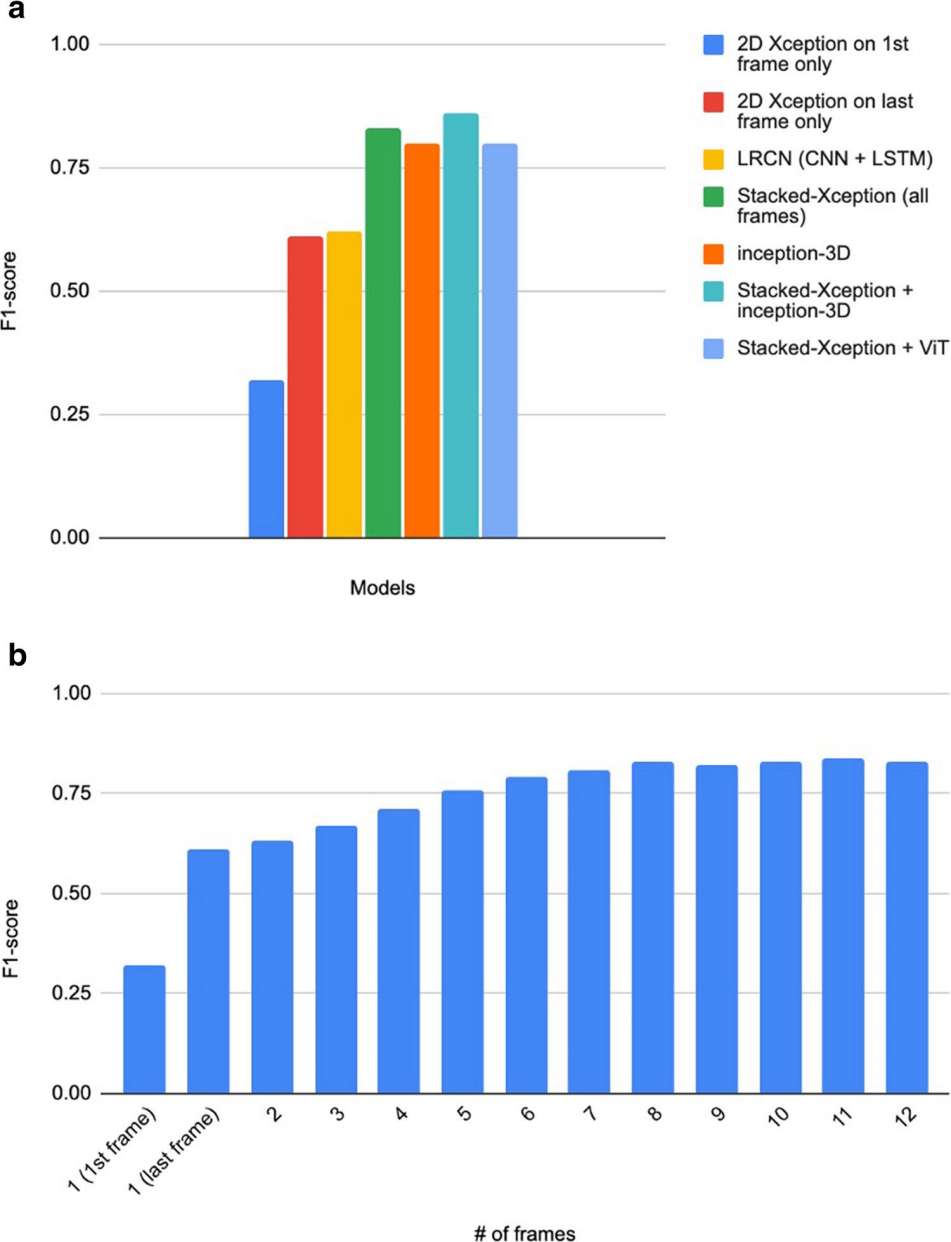
**a** F1 score of different architectures across the Stanford test set. **b** F1 score comparing 2D-only and stacked 2D

We compare stacked-Xception to a fully 3D convolutional approach using Inception-3D. Both architectures were first pretrained on large-scale datasets (ImageNet and Kinetics-600). The confusion matrices on the Stanford hold-out set (Fig. 2b) indicate the weakness of the 3D approach on correctly identifying the M2 subtype. However, the 3D approach does better in predicting the ICAs. The overall F1 score is only higher for the stacked-Xception (0.83 versus 0.8) as shown in Fig. 3a. In Fig. 3a, we jointly train and evaluate an ensemble model that combines the features from both the stacked-Xception and 3D. The F1 score of the resulting model is slightly higher (0.85) than either the stacked or 3D models individually. The last approach is an ensemble of stacked-Xception and a vision transformer (2D on individual frames). In absolute terms, ICA identification is better than with the stacked-Xception and M2 identification is better than the inception-3D. However, the F1 score (0.8) is marginally lower than the stacked-Xception + 3D.

### Post-thrombectomy videos

**Fig. 4:**
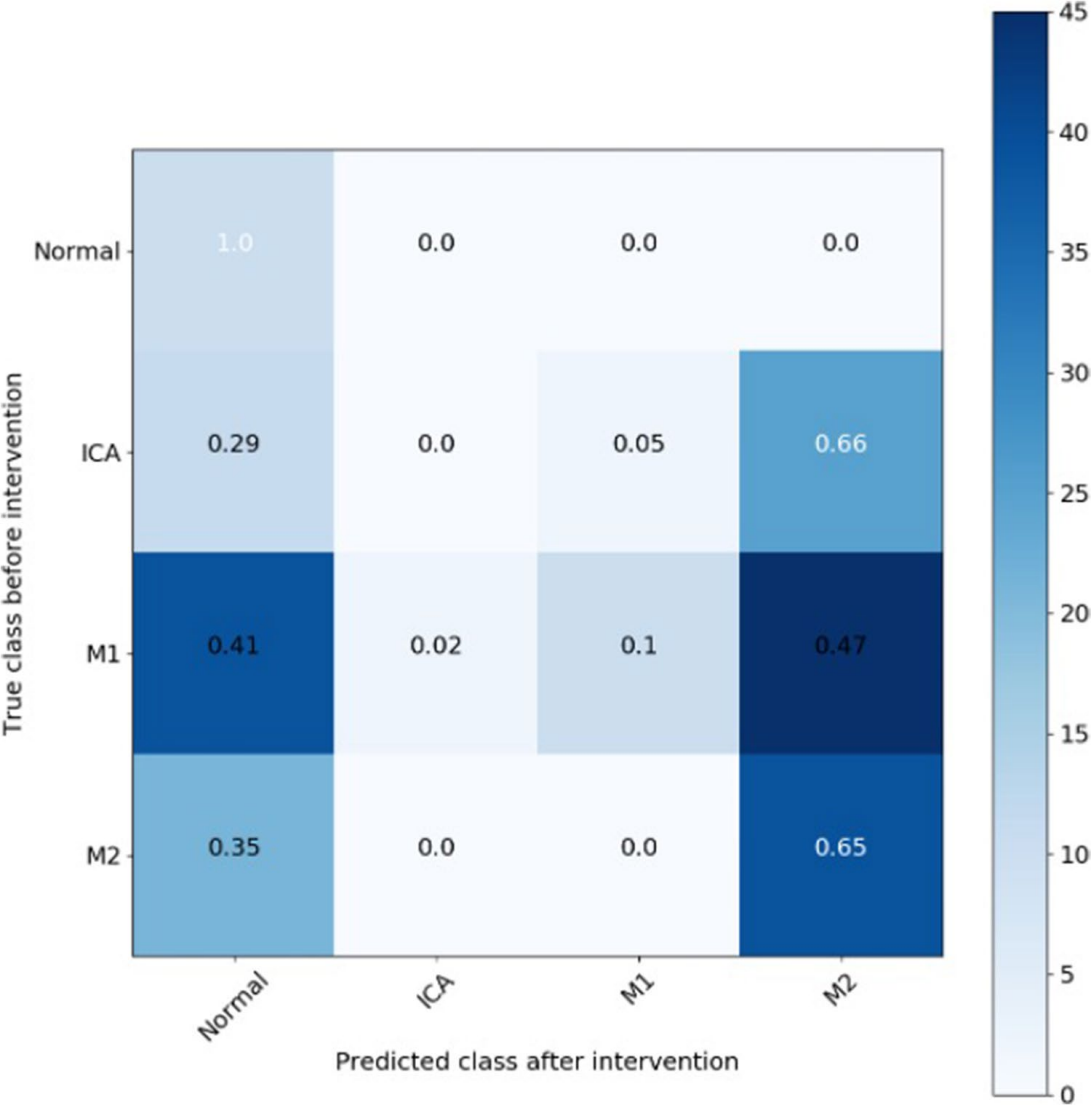
Confusion matrix of the stacked-Xception model predicting videos after intervention in ICA, M1, and M2

Analysing stacked-Xception performance on videos post thrombectomy (*n* = 194), the model identified successful reperfusion with 100% accuracy for ICA occlusions and 88% for M1, and 35% for M2 occlusion (Table 2 and Fig. 4). The model could also perform binary classification of post-intervention videos (ICA, M1, and M2) as having an mTICI of 3 (complete antegrade reperfusion) or < 3 with an AUC of 0.71.

### Grad-cam videos comparing pre- and post-thrombectomy videos

**Fig. 5:**
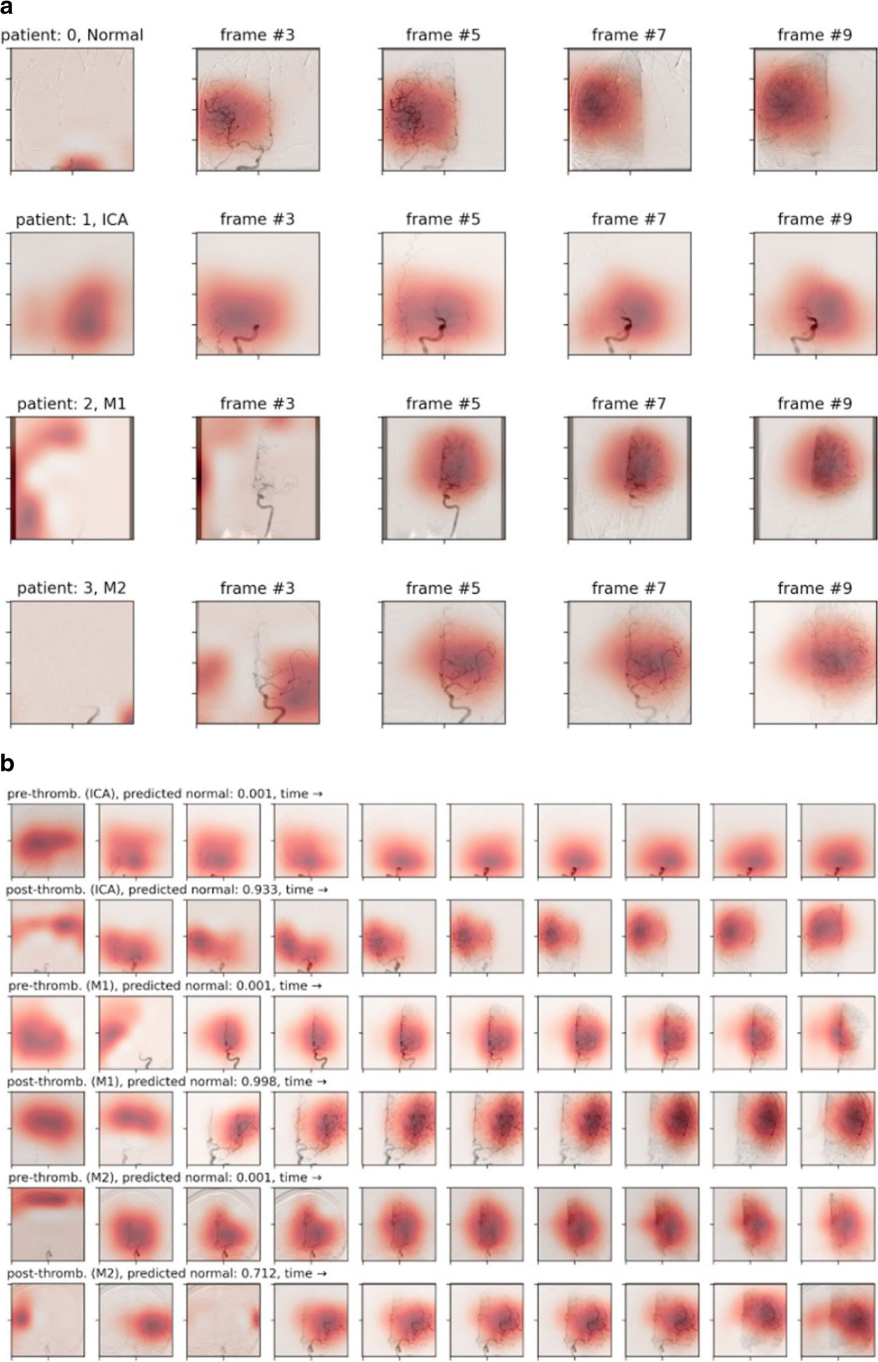
**a** Grad-cam attention maps from the stacked-Xception model on the test set. **b** Grad-cam visualisation of 3 patients with M1 occlusion pre and post treatments

Figure 5a and b shows gradient attention maps of DSA videos from the model trained on the pre-thrombectomy DSAs. The shaded area represents what the model learns to attend to in order to make its prediction. By comparing the post-treatment grad-cam to the pre-thrombectomy video, it is possible to interpret the most important regions to the thrombectomy’s success or failure. The video can be viewed in supplementary material. Finally, grad-cams from the pre-treatment model applied to the post-treatment model give interpretable evidence that our models learn meaningfully relevant features of the thrombectomy.

### Results of external validation

Occlusion identification was achieved with 91.30% sensitivity (71.96 to 98.93%) and 81.82% specificity (48.22 to 97.72%). Accuracy of location classification was 73, 35, and 50% for ICA, M1, and M2, respectively. Location classification was correct to + / − one category in 41 of 44 (93%) of cases. The model identified successful reperfusion with 89% accuracy for ICA occlusions, 88% for M1 occlusions, and 60% for M2 occlusions comparing favourably with the internal data. Results are summarised in Table 2.

## Discussion

Herein, we present a model that inputs video of a cerebral DSA to (1) identify the presence or absence of large vessel occlusion in acute stroke; (2) locate the level of occlusion (terminal ICA, M1, or M2); and (3) assess the effectiveness of thrombectomy.

AI has achieved expert-level performance in radiology, but only in specific narrow domains [16, 17]. More recently, the concept of augmented intelligence has gained traction within the radiology literature [18]. While AI applications hold great promise in healthcare, there is a chasm between academia and the clinical application of models [19]. Acute stroke care has recently been disrupted by the advent of mechanical thrombectomy for large vessel occlusion, which has had a dramatic impact on clinical practise [20, 21]. Future potential clinical utility for models such as ours would include providing decision support via rapid interpretation (pre thrombectomy) to aid detection of more subtle or distal thrombi and automated objective gradation of thrombectomy outcomes (post thrombectomy). “Time is brain” and if residual thrombus could be identified more quickly or if operators could be informed that > 50% antegrade reperfusion has not been obtained within 60 min [22], then there is the potential to improve patient outcomes with such an automated detection model.

There are two additional elements of our research questions that add temporal complexity. The video files have a temporal element as they follow the administration of contrast and the opacification of vessels over time. The temporal element is necessary for correct classification as parts of the vessel may be opacified in certain frames and not others due to the haemodynamic circulation. Considering multiple frames however necessitates adding considerable superfluous information as frames before the contrast has reached the region of interest do not contain diagnostic information. This reduces the signal-to-noise ratio of the input, and while necessary, it makes the task more challenging. Furthermore, there is the interval change aspect whereby we evaluate videos before and after intervention. Development of such models has been deemed “essential to provide meaningful improvements in clinical workflows” [8].

We have explored a diverse set of deep learning architectures for stroke video as shown in Table 3. 2D models (models with only 2 dimensional convolutions) pretrained on ImageNet achieve high classification accuracy in radiology [5, 23]. While they are effective for still imaging, they lack the capacity to incorporate temporal information. Methods such as optical flow are traditionally used [24–26]. Nonetheless, 2D models are effective in scenarios where the region of interest is unique (or concentrated) to a single frame, perhaps even more than 3D approaches. In a DSA video, there can be frames that contain little to no information related to the occlusion itself; noisy or distorted frames in the video can even damage the AI performance. By using 3D CNNs, we can capture full spatial and temporal information, and using 3D CNNs is useful when the number of frames is large. The 3D model size (and number of trainable parameters) is typically larger than a 2D counterpart, as the convolutional kernels have an extra dimension. Nonetheless, for our 3D model, we choose a model that is of the same approximate size as a 2D model (Table 3). High model variance can be alleviated by pretraining on a large-scale video dataset. In our work, we utilised both pretrained and non-pretrained 3D model with Kinetics-600 [27]. The third method we considered is a stacked CNN. This method passes each frame into a 2D CNN, concatenates the features, and mixes at the feature-level across the time dimension. The fundamental difference between the stacked and fully 3D methods is that the stacked CNN lacks joint spatial and temporal representations via 3D convolutions. This has both positives and negatives as while 3D kernels can capture time-dependent continuity of vessel occlusion from frame to frame, these kernels have larger receptive fields (compared to 2D) that can adversely affect the final predictions. For example, a DSA patient video may contain only a few frames with occlusion and many redundant frames with no occlusion. These redundant frames can perturb the final prediction signal. This is demonstrated by the plateau of performance with additional frames in Fig. 3b. Finally, we apply vision transformers (ViTs) [28] that apply layers of multi-headed self-attention and multi-layer perceptrons (MLP) on image patches. ViTs have recently shown strong robustness against image distortions (such as occlusions, permutations) when compared to state-of-the-art CNNs and are less sensitive to colour and texture bias than CNNs [28]. There is growing evidence that CNNs [29] rely mainly on texture (and colour) information rather than shape.

**Table 3:** Qualitative exploration and number of trainable parameters of deep learning architectures for DSA video

Model	Potential advantages	Potential limitations
2D CNN	Simple to use, useful when region of interest is unique to a single frame. Leverages large-scale ImageNet dataset	Lacks temporal dependency
3D CNN	Captures full spatial and temporal dependency, useful when # of frames are large. Leverages large-scale Kinetics video dataset	Typically larger in model size than an equivalent 2D model due to the added kernel dimension
Stacked 2D CNN model	Simple to use (with limited frames), easy to interpret DSA over a few individual frames. Leverages ImageNet pretraining on 2D feature extractors	Feature-level temporal dependency only. There are no joint spatial and temporal dependencies
2D vision transformer	Robust to frame-level distortions, relaxed inductive bias	2D features only Limited training dataset size may limit the final performance

We have investigated numerous deep learning approaches and found both the stacked-Xception and stacked-Xception + inception-3D produced high classification accuracies in tasks 1 and 2 with reasonably low model complexity. Both models were pretrained from large-scale images and video datasets. We have shown that more frames throughout the DSA video are necessary but with limited improvements in F1 score after 8 input frames. By using more than 20 frames, all approaches would require more complexity in computational and memory overheads.

**Fig. 6:**
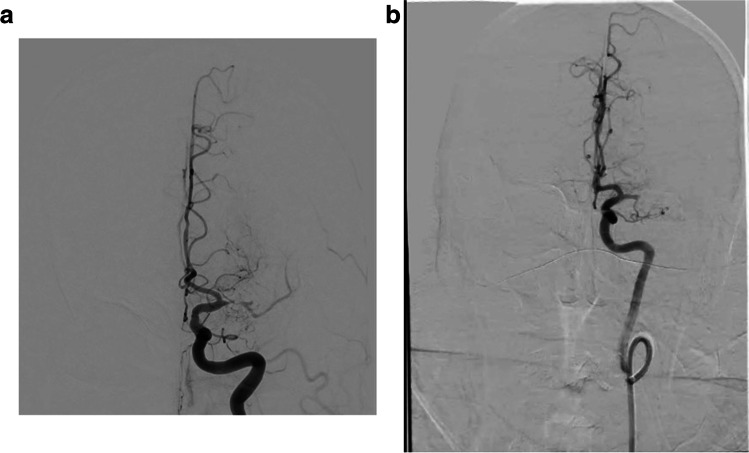
**a**, **b** Illustrative example comparing the difference in the videos between internal and external validation (**a** left: Stanford M1/Pre, **b** right: IU M1/Pre)

We conducted a robust external validation of our primary model to examine its potential for generalizability. The DSA videos from the external site were obtained using different fluoroscopy equipment and stored on different imaging platforms compared to the local data. Furthermore, the technique of obtaining the images is somewhat different as seen in Fig. 6a and b. The local images are centred higher, and thus the region of interest is in a different location in the local and external images making generalizability more challenging. While the overall location accuracy reduced, occlusion localisation was within + / − one category in 95% of cases meaning that the vast majority of miss-classifications were near misses.

Grad-cams in Fig. 5a and b highlight the regions of each frame that are attended to by the trained model. They illustrate that the pattern of attention is different for those videos in different classes. For example, in an M2 occlusion case, the patient’s treatment (Fig. 5b) appears to have been successful as the model classifies this post-thrombectomy video as normal with 71.2% confidence. This has potential clinical utility both in real-time decision support to assess the efficacy of a particular pass and also retrospectively to help standardise TICI grading. Furthermore, we can see that the attention in the example of an ICA occlusion is concentrated more inferiorly than in the case of a normal or M2 study due to the anatomical location of the occlusion. This intuitive observation gives confidence that the model is behaving as we would expect. A supplementary video shows the changing attention map frame by frame. The pre- versus post-thrombectomy grad-cams were computed by taking a model trained to classify pre-thrombectomy DSAs and evaluating the post-thrombectomy DSAs. This was not only to assess the quality of the treatment (from the model’s point of view) but also to provide interpretable evidence that our models learn meaningful features. For example, the model does not attend to the eyes or the skull, where the shapes and textures of non-vessel objects may bias the prediction.

Our results are promising but have limitations. Possibly due to the limited sample size and overfitting, the 3D model trained from scratch performed considerably worse than the 2D, stacked, or 3D models leveraging ImageNet or Kinetics-600 pretraining. Despite the consistent performance on the external validation set, it is important to note that a limited selection of patients within one country are included. However, imaging in acute stroke is quite uniform, and the performance on unseen data demonstrates a degree of robustness in our model that bodes well for future generalizability. We enrolled consecutive patients and externally validated our research with the aim of reduction of selection bias in this study. Three patients’ video files were corrupted and we were not able to include them in our study. This was due to the video conversion process, and hopefully, if this technique becomes more popular, these technical hurdles will be overcome. Our dataset could be interpreted as having bias classes, with 75% of our cases having pathology. To alleviate this, we used sensitivity, specificity, or AUC when reporting results from bias classes. Importantly, the relatively even split between normal, ICA, M1, and M2 classes is in keeping with the population who proceed to thrombectomy [30].

## Conclusion

Our models can successfully identify normal DSA studies from those with ICA, M1, and M2 occlusions, and can classify the outcome of mechanical thrombectomy via mTICI grading. This has the potential to increase the speed, accuracy, and efficacy of stroke care by augmenting performance of neurointerventional radiologists. It could also be used for objective assessment of thrombectomy outcomes. Furthermore, we have addressed a problem with two temporal elements, dynamic video and pre and post-intervention, with a unique application of state-of-the-art methods.

## Supplementary Information

Below is the link to the electronic supplementary material.

Grad Cam video showing the dynamic changing attention map. We can see the region of interest attended to by the model shift from frame to frame. Label 0 are normal cases, label 1 are ICA occlusions, label 2 M1 occlusions and label 3 M2 occlusions.
Supplementary file1 (MP4 1856 KB)

## Data Availability

The data that support the findings of this study are available from the corresponding author(s) upon reasonable request.

## Abbreviations

AI: Artificial intelligence
CI: Confidence interval
CLAIM: Checklist for Artificial Intelligence in Medical Imaging
CNN: Convolutional neural network
DICOM: Digital Imaging and Communications in Medicine
DSA: Digital subtraction angiography
HIPAA: Health Insurance Portability and Accountability Act
ICA: Internal carotid artery
LVO: Large vessel occlusion
MCA: Middle cerebral artery
MLP: Multi-layer perceptron
mTICI: Modified Treatment in Cerebral Ischaemia
PA: Posteroanterior
ViT: Vision transformer

## Declarations

### Guarantor

The scientific guarantor of this publication is KW Yeom.

### Statistics and biometry

One of the authors has significant statistical expertise. No complex statistical methods were necessary for this paper.

### Methodology

• Retrospective

• Experimental

• Multicentre study
